# Microwave-Assisted Extraction of Oleanolic Acid and Ursolic Acid from *Ligustrum lucidum* Ait

**DOI:** 10.3390/ijms12085319

**Published:** 2011-08-18

**Authors:** En-Qin Xia, Bo-Wei Wang, Xiang-Rong Xu, Li Zhu, Yang Song, Hua-Bin Li

**Affiliations:** 1 Guangdong Provincial Key Laboratory of Food, Nutrition and Health, School of Public Health, Sun Yat-Sen University, Guangzhou 510080, China; E-Mails: enqinxia@163.com (E.-Q.X.); boweiwno@163.com (B.-W.W.); 898013628@qq.com (L.Z.); sssongyang@163.com (Y.S.); 2 Key Laboratory of Marine Bio-resources Sustainable Utilization, South China Sea Institute of Oceanology, Chinese Academy of Sciences, Guangzhou 510301, China; E-Mail: xuxr2000@yahoo.com

**Keywords:** microwave, extraction, oleanolic acid, ursolic acid, *Ligustrum lucidum*

## Abstract

Oleanolic acid and ursolic acid are the main active components in fruit of *Ligustrum lucidum* Ait, and possess anticancer, antimutagenic, anti-inflammatory, antioxidative and antiprotozoal activities. In this study, microwave-assisted extraction of oleanolic acid and ursolic acid from *Ligustrum lucidum* was investigated with HPLC-photodiode array detection. Effects of several experimental parameters, such as type and concentration of extraction solvent, ratio of liquid to material, microwave power, extraction temperature and microwave time, on the extraction efficiencies of oleanolic acid and ursolic acid from *Ligustrum lucidum* were evaluated. The influence of experimental parameters on the extraction efficiency of ursolic acid was more significant than that of oleanolic acid (*p* < 0.05). The optimal extraction conditions were 80% ethanol aqueous solution, the ratio of material to liquid was 1:15, and extraction for 30 min at 70 °C under microwave irradiation of 500 W. Under optimal conditions, the yields of oleanolic acid and ursolic acid were 4.4 ± 0.20 mg/g and 5.8 ± 0.15 mg/g, respectively. The results obtained are helpful for the full utilization of *Ligustrum lucidum*, which also indicated that microwave-assisted extraction is a very useful method for extraction of oleanolic acid and ursolic acid from plant materials.

## 1. Introduction

**Figure 1.**
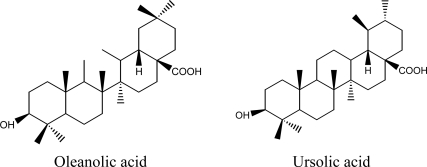
The chemical structures of oleanolic acid and ursolic acid.

*Ligustrum lucidum* Ait is widely distributed in China. The fruit of *Ligustrum lucidum* is a famous traditional Chinese medicine (Nvzhenzi in Chinese), and has been used for prevention and treatment of several diseases, such as diabetes and coronary heart disease [1–5]. It contains a variety of physiologically active compounds, and oleanolic acid and ursolic acid are its main active components [4,6,7]. Both oleanolic acid and ursolic acid have attracted a lot of attention because of their various biological activities, such as anticancer, antimutagenic, anti-inflammatory, antioxidative and antiprotozoal properties [3,8–10]. The chemical structures of oleanolic acid and ursolic acid are shown in Figure 1.

The extraction of active ingredients from *Ligustrum lucidum* can be carried out in various ways, such as maceration extraction, reflux and Soxhlet extraction [7,11,12]. However, these conventional extraction methods are usually time- and solvent-consuming. In recent years, microwave-assisted extraction (MAE) has been developed for the extraction of some important bioactive compounds from plant materials [13–16]. Microwave heats the extraction solvent quickly, and accelerates the desorption process of the targeted compounds from matrix. MAE is a green method with a significant reduction in extraction time and organic solvent consumption [17,18]. However, it was unknown whether the extraction efficiencies of oleanolic acid and ursolic acid from *Ligustrum lucidum* could be improved by the microwave-assisted extraction.

Optimization of extraction conditions is very important, as it can improve the extraction yields of the targeted compounds, and fully utilizes the plant materials [16,19–25]. The crude extract obtained could be used as either components of some complex traditional medicines or the further isolation and purification of targeted compounds. In this paper, microwave-assisted extraction of oleanolic acid and ursolic acid from *Ligustrum lucidum* was studied by HPLC with photodiode array detection. Effects of several experimental parameters, such as type and concentration of extraction solvent, ratio of liquid to material, microwave power and time, as well as extraction temperature, on the extraction efficiencies of oleanolic acid and ursolic acid from *Ligustrum lucidum* were evaluated. The results obtained are helpful for the full utilization of *Ligustrum lucidum*.

## 2. Results and Discussion

### 2.1. Effect of Different Solvents on the Extraction Efficiencies of Ursolic Acid and Oleanolic Acid

**Figure 2.**
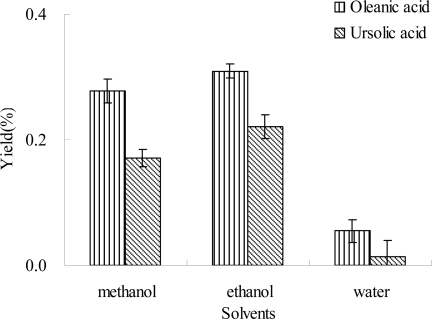
Effect of the different solvents on the extraction yield. Experimental conditions were: the ratio of liquid to material, 10:1 (mL/g); microwave power, 300 W; extraction temperature, 40 °C; and extraction time, 30 min.

In the present study, effects of several solvents, such as ethanol, methanol and water on the extraction efficiencies of ursolic acid and oleanolic acid were evaluated respectively. Seen from Figure 2, the yields of oleanolic acid and ursolic acid followed the analogical trend among all the tested solvents, and the yield of oleanolic acid was higher than that of ursolic acid at the same experimental condition. The yields of oleanolic acid and ursolic acid extracted by ethanol were the highest (3.0 ± 0.12 and 2.1 ± 0.18 mg/g), and slightly lessened by methanol (2.8 ± 0.19 and 1.7 ± 0.14 mg/g). However, the yields of oleanolic acid and ursolic acid extracted by water were the lowest. In fact, ethanol or methanol was mainly chosen as an extraction solvent to extract ursolic acid and oleanolic acid from plant materials in the literature [26–28]. The difference between the yields of ursolic acid and oleanolic acid extracted by water and ethanol or methanol was concurred with previous reports [29,30]. The extraction of organic component from plant materials is directly related to its polarity matching to the extraction solvent [31,32]. As is known from the chemical structures of ursolic acid and oleanolic acid (Figure 1), they should possess relatively low polarities. Thus, the polarities of ethanol and methanol were more matched to those of ursolic acid and oleanolic acid than that of water, which results in higher yields. In addition, methanol is a more toxic solvent than ethanol to human beings and the environment. Therefore, ethanol was selected as an extraction solvent for the sequent study.

### 2.2. Effect of Ethanol Concentration on the Extraction Efficiencies of Ursolic Acid and Oleanolic Acid

**Figure 3.**
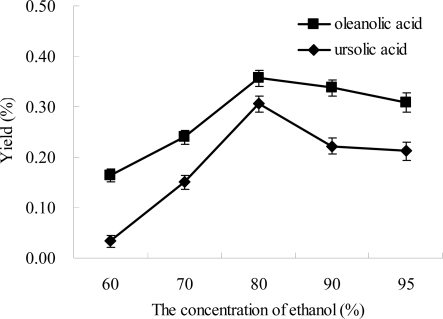
Effect of ethanol concentration on the extraction yield. Experimental conditions were: the ratio of liquid to material, 10:1 (mL/g); microwave power, 300 W; extraction temperature, 40 °C; and extraction time, 30 min.

Although water showed the lowest extraction efficiency (Figure 2), it is a nontoxic and inexpensive solvent. In addition, water has widely been applied for extraction of bioactive compounds in Chinese traditional medicines, and sometimes the extraction efficiency could be improved using a mixture of ethanol and water [33]. Thus, effect of concentration of ethanol on the extraction efficiencies of ursolic acid and oleanolic acid from *Ligustrum lucidum* was evaluated. The results are shown in Figure 3, and a similar trend was observed for the yields of ursolic acid and oleanolic acid with the increase of ethanol concentration. When the concentration of ethanol increased from 60% to 80%, the yields of oleanolic acid and ursolic acid significantly increased, followed by a slight decrease with further increase of ethanol concentration from 80% to 95%. The yields of oleanolic acid and ursolic acid simultaneously reached the maximum values at 80% ethanol, which were 3.6 ± 0.18 and 3.1 ± 0.16 mg/g for oleanolic acid and ursolic acid, respectively. The results indicated that 80% ethanol was suitable for the extraction of oleanolic acid and ursolic acid from *Ligustrum lucidum*. The yields of oleanolic acid and ursolic acid extracted by 80% ethanol was significantly higher than those extracted by 100% ethanol, which were 3.1 ± 0.11 and 2.2 ± 0.19 mg/g for oleanolic acid and ursolic acid, respectively (Figure 3). These results supported previous findings that a mixture of ethanol and water was the best solvent for the extraction of triterpene acids from different natural products [30]. Therefore, in the subsequent experiments, 80% ethanol was used.

### 2.3. Effect of the Ratio of Liquid to Material on the Extraction Efficiencies of Ursolic Acid and Oleanolic Acid

**Figure 4.**
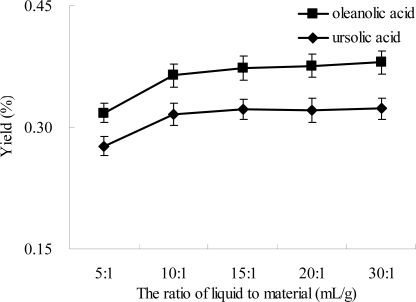
Effect of the ratio of liquid to material on the extraction yield. Experimental conditions were: extraction solvent, 80% ethanol; microwave power, 300 W; extraction temperature, 40 °C; and extraction time, 30 min.

Effect of the ratio of liquid to material on the extraction yield of oleanolic acid and ursolic acid was investigated. The results are displayed in Figure 4, and an analogical behavior of oleanolic acid and ursolic acid was observed. When the ratio of liquid to material increased from 5:1 to 15:1, the yields of oleanolic acid and ursolic acid increased. When the ratio of liquid to material increased from 15:1 to 40:1, the yields remained almost unchanged. The yields of oleanolic acid and ursolic acid simultaneously reached the maximum values at 15:1 of the ratio of liquid to materials. Generally, the large ratio of liquid to material dissolves components more effectively, and results in an enhanced extraction yield [34,35]. Therefore, the ratio of liquid to material at 15:1 was chosen as the optimal condition for subsequent experiments.

### 2.4. Effect of Microwave Power on the Extraction Efficiencies of Ursolic Acid and Oleanolic Acid

**Figure 5.**
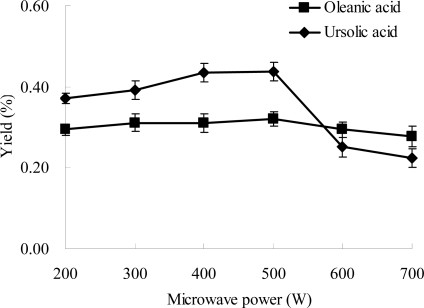
Effect of microwave power on the extraction yield. Experimental conditions were: extraction solvent, 80% ethanol; the ratio of liquid to material, 15:1; extraction temperature, 40 °C; and extraction time, 30 min.

Effects of microwave power on the extraction yield of oleanolic acid and ursolic acid were investigated. The results are shown in Figure 5, and different behavior observed for oleanolic acid and ursolic acid. The yield of oleanolic acid remained almost unchanged from 200 to 700 W, with a slightly high value at 500 W. On the other hand, the yield of ursolic acid markedly increased from 200 to 500 W, and then obviously decreased from 500 to 700 W. The highest yield was obtained at 500 W (Figure 5). Maybe, the different behavior was because of the differences in their chemical structures as well as their combination power with other molecules in this plant. The microwave can accelerate the extraction process for desorption of the targeted compounds from matrix at low power, and may induce the decomposition of some target molecules at high power [36]. Therefore, 500 W of microwave power was used in sequent experiments.

### 2.5. Effect of Extraction Temperature on the Extraction Efficiencies of Ursolic Acid and Oleanolic Acid

**Figure 6.**
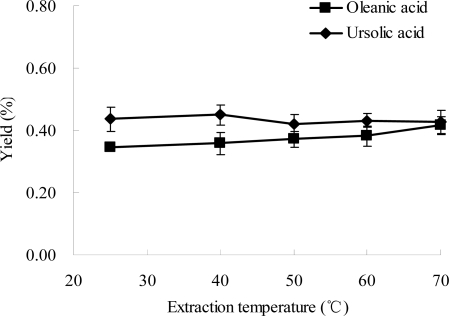
Effect of extraction temperature on the extraction yield. Experimental conditions were: extraction solvent, 80% ethanol; the ratio of liquid to material, 15:1; microwave power, 500 W; and extraction time, 30 min.

Effect of extraction temperature on the yields of oleanolic acid and ursolic acid was evaluated. The results are shown in Figure 6. When the temperature increased from 25 to 70 °C, the yield of oleanolic acid slightly increased, and the yield of ursolic acid remained almost unchanged. Generally, the extraction temperature could influence the recovery of bioactive ingredients during liquid-solid extraction [31]. Increasing temperature of extraction medium can increase the diffusivity of the solvent into cells and enhance the desorption and solubility of compounds from the cells, which results in the dissolution of components [37,38]. Although some bioactive compounds from plants could be decomposed at a high temperature [38,39], the yields of oleanolic acid and ursolic acid did not decrease at 70 °C. The results indicated that oleanolic acid and ursolic acid are thermally stable components up to 70 °C. Thus, 70 °C was chosen as the optimal extraction temperature.

### 2.6. Effect of Extraction Time on the Extraction Efficiencies of Oleanolic Acid and Ursolic Acid

**Figure 7.**
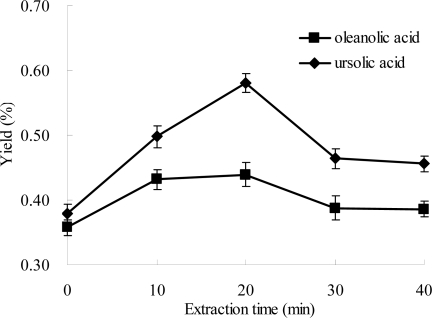
Effect of extraction time on the extraction yield. Experimental conditions were: extraction solvent, 80% ethanol; microwave power, 500 W; and extraction temperature, 70 °C.

Effect of extraction time on the yields of oleanolic acid and ursolic acid was investigated. The results are shown in Figure 7, and a similar trend was observed for oleanolic acid and ursolic acid. The yield of ursolic acid markedly increased from 0 to 20 min, and then obviously decreased from 20 to 30 min, which followed with an almost unchanged status until 40 min. The yield of oleanolic acid obviously increased from 0 to 10 min, and kept almost unchanged from 10 to 20 min, and then markedly decreased from 20 to 30 min, followed by an almost unchanged value until 40 min. The yields of oleanolic acid and ursolic acid simultaneously reached the maximum values at 20 min. The results indicated that microwave might accelerate dissolution of target compounds from plant cell wall in a short time, which was a huge advantage of microwave-assisted extraction compared to conventional extraction methods [11,40]. Both oleanolic acid and ursolic acid might be degraded over a longer time, which would induce decreased yields. Thus, 20 min was selected as the optimal extraction time.

Under the optimal conditions, the yields of oleanolic acid and ursolic acid were 4.4 ± 0.20 mg/g and 5.8 ± 0.15 mg/g, respectively. Seen from Figure 2 to Figure 7, the yields of ursolic acid and oleanolic acid increased with the optimization of experimental parameters, and the increase of ursolic acid was more significant than that of oleanolic acid, which was almost 1.5 times higher under optimal conditions than in the first step of experiments. It indicated that the optimization of extraction procedure was very important to the full utilization of *Ligustrum lucidum*.

## 3. Experimental Section

### 3.1. Chemicals

Oleanolic acid and ursolic acid were bought from Siyi Biotechnology Company (Chengdu, China). Acetonitrile, ethanol and methanol were HPLC grade and purchased from Merck (Germany). Acetic acid was bought from Tianjin Chemical Reagent Company (Tianjin, China). Deionized water was used throughout the experiment.

The stock solutions of oleanolic acid and ursolic acid (10 mg/mL) were prepared in methanol, and stored at 4 °C. The calibration standards (5–250 μg/mL for ursolic acid and 10–500 μg/mL for oleanolic acid) were prepared from the stock solution by the serial dilution of methanol.

### 3.2. Instruments

The microwave-assisted extraction was carried out in an X-100A microwave extraction device (Xianghu Instrumental Company, Beijing, China) with a microwave power of 1000 W and a monitor of temperature as well as microprocessor programmer software to control performance parameters of the microwave device, *i.e*., microwave power, temperature and running time.

### 3.3. Plant Material and Sample Treatment

The fruit of *Ligustrum lucidum* (with humidity of 2.31%) was purchased from a drugstore in Guangzhou, Guangdong Province, China, and ground into powder in a knife mill, and then stored at 4 °C in a refrigerator.

The powder (1 g) was accurately weighed, and placed in a capped glass tube, and then mixed with an appropriate amount of extraction solvent. After soaking for 30 min that permitted solvent to wet plant material, the tube with sample was immersed into water in the microwave device, and irradiated for the pre-set extraction temperature and time. After extraction, the sample was centrifuged at 9600 g for 10 min, and then the supernatant was collected and diluted for HPLC analysis.

### 3.4. HPLC Analysis

A Waters (Milford, MA, USA) 1525 binary HPLC pump separation module with an auto-injector (20 μL) and a Waters 2996 photodiode array detector was used. An Agilent Zorbax Extend-C18 column (250 mm × 4.6 mm, 5 μm) was used. The mobile phase was acetonitrile and 0.5% acetic acid in water (90:10, v/v) with a flow-rate of 0.8 mL/min, and the column temperature was kept at 27 °C [27,28]. The UV spectra were recorded between 190 and 400 nm for peak characterization, and the wavelength of detection was set at 210 nm. The peak area was used to calculate the amount of oleanolic acid and ursolic acid from the standard curve.

All the experiments were conducted in triplicate, and the average values ± SD (standard deviation) were reported.

## 4. Conclusions

A microwave-assisted extraction method has been developed for the extraction of oleanolic acid and ursolic acid from *Ligustrum lucidum*. Effects of several experimental parameters on the extraction yields of oleanolic acid and ursolic acid have been evaluated, and the influence of experimental parameters on the yield of ursolic acid is more significant than that of oleanolic acid. The optimal extraction conditions were as follows: extraction solvent, 80% ethanol; the ratio of liquid to material, 15:1; microwave power, 500 W; extraction temperature, 70 °C; extraction time, 20 min. Under the optimal conditions, the yields of oleanolic acid and ursolic acid were 4.4 ± 0.20 mg/g and 5.8 ± 0.15 mg/g, respectively. Microwave is a powerful tool, which can efficiently improve the extraction performance of oleanolic acid and ursolic acid. The crude extract could be used as either components of some complex traditional medicines or for further isolation and purification of oleanolic acid and ursolic acid. The results obtained are helpful for the full utilization of *Ligustrum lucidum*.
